# A Step towards Understanding and Tackling Health Inequalities: The Use of Secondary Prevention Services and the Need for Health Promotion in a Rural Setting

**DOI:** 10.3390/ijerph18168492

**Published:** 2021-08-11

**Authors:** Monika Karasiewicz, Ewelina Chawłowska, Agnieszka Lipiak, Barbara Więckowska

**Affiliations:** 1 Department of Preventive Medicine, Poznan University of Medical Sciences, 60-781 Poznan, Poland; ewierz@ump.edu.pl (E.C.); alipiak@ump.edu.pl (A.L.); 2 Department of Computer Science and Statistics, Poznan University of Medical Sciences, 60-806 Poznan, Poland; barbara.wieckowska@ump.edu.pl

**Keywords:** health promotion, healthcare disparities, health inequalities, rural health services, general practice, cancer screening, diagnostic tests, out-of-pocket payments, health literacy, farmers, community-based participatory research

## Abstract

Poland has recently intensified its health promotion in an effort to extend healthy life expectancy and reduce health inequalities. Our aim was to reach a deprived rural population, increase its health literacy, and explore its use of and barriers to cancer screening and public health care. A CBPR study was conducted in one of the poorest districts in Wielkopolska region, Poland, among 122 beneficiaries of health education workshops. A self-developed questionnaire was used. The reported barriers to participation in cancer screening included: lack of time, lack of need, or feeling healthy (32.8%); long waiting times (17.2%); fear of costs (9%). Physicians seldom recommended screening to their patients. Only 7.4% of respondents had ever received dermatoscopy. Among women, 18.2% did not perform any breast exams and 25% had never had smear tests. Diagnostics was often financed out of pocket (thyroid ultrasound = 58.1%; smear test = 48.5%; breast ultrasound = 36.8%). The health system needs mentioned by participants included better access to physicians (65.6%), promotion of free screening tests (54.9%), and access to public health programmes (22.1%). There is an urgent need to translate national strategies into action. Health promotion and better access to care must become priorities in deprived areas, while primary care providers should become key figures in delivering these services.

## 1. Introduction

A high prevalence of health problems determined primarily by lifestyle-related factors can be observed in Poland [1,2,3]. Many of them are well known, and some can be modified by changes in health behaviour or controlled through screening. Even in very serious diseases such as cancer, the risk can be effectively reduced if certain preventive steps are taken [4]. This is why the current Polish regulations provide for strengthening the role of health prevention in primary healthcare (PHC), with the main focus being on chronic diseases. According to the Act on Primary Health Care of 27 October 2017 [5], the activity of primary healthcare providers in the area of preventing chronic diseases will be increasingly supported and intensified. One of the means to that end is a coordinated care programme currently being piloted. It consists of preventive health checks in patients aged 20–65 years [6]. Other preventative strategies are delineated into two consecutive National Health Programmes (for the years 2016–2020 and for the years 2021–2025), which aim to prolong healthy life expectancy, improve health-related quality of life, and reduce health inequalities [7,8]. There are also hopes that the National Cancer Control Strategy for the years 2020–2030 will bring systemic change to Polish oncology and reorient it towards prevention [9]. In addition, large-scale state-funded public health programmes (PHPs) are implemented throughout the country by the National Health Fund (NFZ) and the Ministry of Health [10], while smaller-scale PHPs are run by local authorities [11,12]. All of these efforts are of paramount importance because Poland has one of the least resilient health systems in the European Union, as evidenced by higher than average mortality from preventable and treatable causes, lower than average survival rates for cancers, and unsatisfactory screening attendance rates [1]. Other challenges faced by the Polish health system include the low quality of care, long waiting times, health personnel shortages, and a relatively high share of out-of-pocket payments [1,13].

The available Polish, European and global research shows health inequalities between populations depending on where they live. The degree of inequalities varies significantly within Europe, and there are a number of models that attempt to capture this variation and embrace the numerous determinants of inequalities [14,15,16,17]. One of the determinants is rural vs. urban residence—it has been demonstrated that rural inhabitants have limited access to health care [18,19,20,21,22] and attend specialist care less often than city dwellers [20,23]. Rural populations have higher prevalence of certain behavioural risk factors [18,19,24,25] and tend to be more difficult to reach with health-promoting messages [18]. They often face systemically limited access to preventative healthcare. For instance, since the majority of Polish farmers are self-employed, they are not subject to obligatory periodic health checks available through occupational health services [26]. Additionally, local PHPs in Poland are significantly less often implemented in rural areas—districts at higher risk of social deprivation and with higher general mortality rates [2].

It seems that special attention should be paid to exploring and responding to the challenges of promoting health in highly deprived areas. In Poland, deprivation can be measured with the so-called district deprivation index, which comprises inhabitants’ income, employment, living conditions, education, and access to goods and services [27]. An example of such a deprived area is Koło district—one of the two districts in Wielkopolska region where high deprivation risk coincides with a high general mortality, lack of access to local PHPs [2], and unsatisfactory attendance rates for mass cancer prevention programmes [28]. As our study site, we chose one of the communes in Koło district (commune; Polish: *gmina*—a small administrative unit), located far from urban areas. According to official analyses, the commune ranks among the most disadvantaged areas in the whole Wielkopolskie province regarding access to education, healthcare, and public utilities [29,30]. The population of the commune (6353 people in 2018) has significantly lower education levels than general Polish population [31]. The inhabitants have limited access to public specialised health care—the only NFZ-financed specialist care provider in the area is a dental clinic [29]. The commune has some beautiful landscapes and tourist attractions, although apart from a few pedestrian and cycling routes, health-supporting infrastructure is scarce. There is a well-developed road network for car traffic but only 1.1 km of cycle paths [32] and 1.4 km of pavements outside the biggest town in the commune [29]. The only freely accessible sports facilities are the playgrounds and sports fields located at and managed by local schools [29]. All of these factors make the setting hardly conducive to an active and healthy lifestyle. The commune inhabitants have poorer health outcomes than the general population. In 2015–2017, life expectancy in the district was in the lowest range in Poland for men (70.2–71.9 years) and in the third lowest range for women (81.0–81.4 years) [2]. In 2019, general mortality in the commune (12.18 per 1000 population) was much higher than in Wielkopolskie province (9.8) and in Poland (10.7) [31].

Having considered all of the above factors, we chose the commune as a site for a field study and an intervention seeking to reduce health inequality and explain its underlying causes, with a focus on barriers to health prevention. The main aim of the study was to analyse the use of and barriers to cancer screening and public health services, including the role of the general practitioner (GP) and the share of out-of-pocket payments, among rural inhabitants of one of the districts with the highest deprivation risks in Wielkopolska region. We also wanted to identify the main health promotion needs in the study group and to find the key focus areas that needed improvement.

To achieve these goals, we determined the study respondents’ self-perceived health, as well as reported health problems, cancer screening attendance, and barriers to attendance. Furthermore, we explored associations between the variables above and the following eight factors: age, gender, education, professional status, occupation, economic status, attitude to cancer screening recommended to a given age group, and family history of cancer. In addition, we asked how often the respondents participated in selected screening and diagnostic tests and how they financed them. Finally, we enquired whether the respondents’ general practitioners (GPs) ever suggested the need for taking part in screening or diagnostics.

Our objective was also to check whether the observations made in our study group were consistent with findings from other studies and whether our results would reflect the average results in the general Polish population. We wanted to join a discussion on health inequalities and their determinants. We attempted to determine if and to what extent the results from a disadvantaged local population would reveal the weaknesses of the health system, while delivering a health promotion intervention to address some of these weaknesses.

## 2. Materials and Methods

A community-based participatory research (CBPR) study was carried out in a commune located in Koło district, Poland, from July to December 2018. Data used for analysis were collected during a cycle of 7 health education meetings held every two weeks in rural community centres throughout the commune. The meetings were directed to both male and female adult inhabitants of the commune who agreed to participate; age over 18 years and consent to participate were the only inclusion criteria. The agenda of the meetings included conducting a survey, followed by workshops on the methods of reducing cancer risk promoted by the European Code Against Cancer [4]. Among other things, the participants were taught how to calculate their BMI and trained how to perform breast self-exams. They also received basic guidance on how to navigate the Polish health system.

The survey was anonymous and conducted prior to the workshop. Participation was voluntary. Convenience sampling was used. Informed consent was obtained from all participants. The questionnaire used in the survey had been developed in the Department of Preventive Medicine of the Poznan University of Medical Sciences, Poland. It included sociodemographic questions (gender, age, place of residence, education, occupation, professional status, economic status), as well as 24 health-related closed-ended questions, with 9 of these allowing multiple answers. The health-related part aimed to determine the participants’ self-perceived health, main health problems, frequency of participation in selected screening and diagnostic tests, and sources of financing for the tests, as well as health system needs, which according to participants, should be considered in the future health policy of the commune.

Statistical analyses were carried out using PQStat v1.6.8 software. The *p* values below 0.05 were considered significant. Various statistical tests were performed depending on the scale applied, model of analysis used, and the kind of associations searched for. To compare multiple independent groups with ordinal scales, we used non-parametric Kruskal–Wallis ANOVA with a post hoc Dunn–Bonferroni test. The dependence between ordinal scales was tested by analysing Spearman’s rank correlation coefficient; the significance of the test as well as the strength and direction of the monotone relationship (r value) were calculated. The dependence between an ordinal scale and a dichotomous variable was checked with a chi-squared test for trends. We performed either Pearson’s chi-squared test or Fisher’s exact test (when the Cochran’s rule for using chi-squared tests was violated) in order to test the relationships between variables of nominal scales. Any possible differences in group sizes resulted from the fact that ‘I don’t know’ or ‘I don’t remember’ answers were not shown in the tables.

## 3. Results

### 3.1. Sociodemographic Characteristics

The study group comprised 122 people aged from 18 to 78 years (mean age 49.1 ± 15.9 years), i.e., 2.3% of the commune’s adult population. There were 88 women (72.1%; mean age 49.3 ± 16.6 years) and 34 men (27.9%; mean age 48 ± 14.5 years), who were living mostly in rural areas (86.1%). Only 13.9% lived in a small town (population of under 5000). Regarding cancer in the family, 54.1% of the participants had a family history of the disease, 36.9% had no such history, and 9% were not sure. The most numerous group in terms of education contained the participants with secondary (35.3%) and vocational education (26.2%). A majority were employed (54.9%). ‘Farmer’ was a present or past occupation for 53.3% of the participants. The reported economic status was mostly good (58.2%) or average (32.8%). The details of the participants’ sociodemographic status are presented in Table 1.

### 3.2. Health Status

The majority of the participants had weight problems. According to BMI measurements, 74 people (60.7%) were obese or overweight (41% overweight, 14.8% class I obesity, 4.9% class II obesity), while 1.6% were underweight. Self-perceived health was average (49.2%) or good (36.9%). The higher the age, the worse the self-perceived health was (r = −0.55; *p* < 0.0001). The health status was also determined by education (r = 0.26; *p* = 0.0034) and reported economic status (r = 0.33; *p* = 0.0002), but not by occupation, although farmers’ health was on the whole poorer (*p* = 0.0605). The respondents with good and very good self-perceived health tended to attend screening less often (*p* = 0.0055). Family history of cancer did not statistically differentiate the participants’ BMI or health status (see Table 2).

The main reported health problems in order of prevalence were musculoskeletal system problems (53.3%), cardiovascular system problems (27%), headaches (18%), obesity (11.5%), digestive system problems (10.7%), allergies (8.2%), diabetes (6.6%), asthma (4.9%), and addictions (4.1%); 8.2% reported having other ailments, while 6.6% had none. Although obesity was found in 19.7% of the participants, it was not perceived as a health problem by over two-thirds of the obese participants.

### 3.3. Participation in Secondary Prevention

When asked if they looked after their health, the participants usually answered ‘rather yes’ (71.3%). The affirmative answers were positively correlated with economic status (r = 0.25; *p* = 0.0065) and were more frequent in the participants who attended screening tests (*p* = 0.0047). Less than half (43.4%) of our respondents said that they attended screening tests. Attendance was significantly more often reported by women (*p* = 0.0025) and by the retired (*p* = 0.0005) and increased with age (*p* < 0.0001); however, it should be pointed out that one-third of the retired said that they did not attend screening. Non-attendance was most common among those with higher (58.3%) and vocational education (53.1%). While relatively young age probably played a role in the first group (mean age 35.17 ± 9.1 years), it did not matter in the second (mean age 53.1 ± 15.3 years). Attendance was also significantly associated with occupation (*p* < 0.0001), being the lowest among other manual workers (15%) and the highest among health professionals (100%). In this respect, health professionals were significantly different from all other occupational groups except teachers and educators (*p* = 0.0549). Every second farmer (50.8%) reported taking part in cancer screening. The participants with family history of cancer tended to take part in screening more often, even though the tendency was not statistically significant (*p* = 0.0585). The reasons for non-attendance in the non-attending group were lack of time and/or lack of need and/or feeling healthy (32.8%), long waiting times to see specialists (17.2%), and fear of costs (9%). The importance of the last barrier tended to increase with age (*p* = 0.0901), although again the trend did not show statistical significance. The better educated the participants were, the more often they mentioned their GPs’ reluctance (*p* = 0.0222) to refer patients for screening tests as the main reason for non-attendance (see Table 3).

We also asked our participants (n = 122) about the frequency of undergoing selected diagnostic tests, together with the frequency of such tests being suggested by a GP, the source of financing for the tests (public or only out of pocket), as well as the frequency of and the reasons for testing (see Table 4).

Just as we had expected, the most common test was a chest X-ray—65.6% of the participants had taken it at least once, while 57.5% of this group had been suggested to take the test by a GP. Most had the radiograph less often than every 5 years or once in a lifetime, with almost exclusive public financing. It was usually performed as part of a check-up, to confirm or rule out a diagnosis, or for ailments or pains. At least one abdominal ultrasound was performed in 54.1% of the respondents, thyroid ultrasound in 25.4%, smear test in 75% of women, mammography in 80.9% of women of the appropriate age, and breast ultrasound in 43.2% of women. The least common test was dermatoscopy (7.4%). Smear tests were rarely recommended by GPs. The most frequently performed tests paid out of pocket were thyroid ultrasounds (58.1%), smear tests (48.5%), and breast ultrasounds (36.8%). The details of all the tests can be found in Table 4.

In addition, we asked our female participants how often they performed breast self-exams. Only 63.6% of women stated that they had ever done so. Among the performing women, 44.6% did it once a month, 33.9% once every 3 months, 14.3% once every 6 months, and 7.1% even less often. Only 41.1% of the performers had ever been suggested to do the exams by their GPs. In addition, it follows from our calculations that 35.2% of the female participants had never had any form of breast imaging, while 18.2% had received neither an imaging test nor a self-exam.

### 3.4. Health System Needs

When asked about the needs regarding the whole health system and the local health policy, our respondents indicated better access to physicians, including specialists (65.6%), promotion of free screening tests (54.9%), organisation of health-related classes or workshops (23.8%), planning and implementation of PHPs (22.1%), development of health-supporting and health-promoting infrastructure (20.5%), setting up a health committee at the commune (16.4%), and setting up and development of support groups (11.5%).

## 4. Discussion

### 4.1. Health Inequalities in the Study Group

Nowadays, the solutions for reducing and eliminating health inequalities are being widely discussed around the world. Social determinants of health inequalities have become increasingly analysed since the establishment of the WHO’s Commission on Social Determinants of Health in 2005 [33]. In Europe, a rich source of knowledge on social inequalities in health and their determinants is the European Social Survey. It can be used to guide the development of equitable health policies [16], including those related to cancer prevention. A number of international efforts have been made to communicate recommendations and promote best practices in this area, such as the iPAAC Contest of Best Practices tackling social inequalities in cancer prevention [34], or the CanCon’s Policy Paper on Tackling Social Inequalities in Cancer Prevention and Control for the European Population [35].

We would like to join the discussion about the methods of tacking health inequalities by showing an urgent need for supporting PHC in deprived areas so that it could increase health chances of their inhabitants in a more effective way. The above data present the health situation and screening behaviours in a mostly rural population inhabiting a commune located far from big cities, within the 20% of the areas with the highest deprivation risk in Wielkopolskie province. Such social inequalities may translate into the health status of disadvantaged populations, which is why we would like to present the current gaps in the Polish healthcare system as exemplified by one of its weakest parts. It should be noted that the inhabitants we reached, despite having worse epidemiological rates and unfavourable social determinants, did not have access to other health promotion initiatives or to local PHPs that could fill the gaps in access to health services. They had to rely on the care offered as part of the NFZ-financed services, with all its limitations.

Given the data presented here as well as in the available research, we are speculating on the reasons for health inequalities, determinants of the low attendance at mass cancer screening programmes [28], and the low life expectancy in the years 2015–2017 in the studied area [2]. The analysis of occupational diseases among farmers in Poland and a preliminary assessment of PHPs available to rural populations revealed enormous health neglect in adult rural residents, which may result, among other factors, from difficulty accessing early mass health prevention and specialised care [26,36]. The neglect was not unnoticed by our study participants—one-fifth of them indicated the need for PHPs, while over 60% indicated the need for better access to physicians, including specialists.

### 4.2. Health Status

Regarding the self-perceived health status in the Polish population, ‘very good’ and ‘good’ ratings were reported by 59.2% of Poles aged 16 years and over in the 2018 EU-SILC study [37] and by as many as 62.7% of Polish adults in the 2014 EHIS study [38]. Our study group perceived their health status as being worse than the general Polish population, with only 45.9% ‘very good’ and ‘good’ ratings. Our data supported the association between perceived good health and reported economic status, age, education, professional status, and cancer screening attendance. Some of these associations were described elsewhere [1,20,21]. The most common condition in our group was musculoskeletal system problems. This confirms earlier observations indicating that the prevalence of such ailments is underestimated and constitutes a serious clinical and social problem in rural populations [26]. The health problem reported by only 11.5% of our participants but, nota bene, actually found in 19.7%, was obesity; a further 41% of the participants were overweight. While the results in our study group fall within the ranges quoted by other sources for the general Polish population [2], it should be noted that our participants often failed to perceive overweight or obesity as a personal health problem. Alarmingly, in high-income and highly industrialised countries, including Poland, BMI tends to be higher in rural areas, especially among women [2,39]. Our findings show that more effective steps should be taken to increase awareness of the causative relationship between overweight or obesity and a number of diseases, including cancers [4] and other conditions reported by our respondents.

### 4.3. Participation in Secondary Prevention

Patients’ knowledge of the importance of secondary prevention and the resulting regular attendance in health checks and screening are known to be among the most important factors decreasing health risks. Among our participants, 43.4% of the whole study group and 54% of those aged 45–64 years reported participating in diagnostic tests for cancer. We did not find any associations between the reported participation rates and the respondents’ education or socioeconomic status. Our findings are consistent with previous studies on mass cancer screening in women [40,41], which did not show any differences in attendance between groups of varying education and socioeconomic status. It seems that some mass screening programmes give equal opportunities to diverse subpopulations and decrease social and economic inequalities [40]. It may also suggest that our patients, when asked about participation in diagnostic tests for cancer, associated the tests primarily with the available mass screening programmes. In contrast, according to a 2012 study from Wielkopolska region, attendance did increase with education and availability of screening programmes, being the lowest among the less educated women and among rural residents. Low attendance was also associated with the insufficient knowledge of screening programmes and the absence of alarming symptoms [42]. Given the fact that the main barriers to attendance in our study group were lack of time, lack of need, or feeling healthy (32.8%), the participants’ knowledge of indications for secondary prevention (age, occupational risks, smoking, family history of cancer, alarming symptoms) and the resulting perceived risk might have also been low.

In our study, we did not find any statistically significant differences in attendance rates between persons with and without family history of cancer, although we observed a tendency for higher attendance in the former group. Much of the available research shows that cancer in the family tends to increase cancer screening participation and indicates that the reasons include stronger encouragement from physicians and stronger personal motivations [41,43].

Another area we explored was the frequency of participating in selected diagnostic tests at least once in a lifetime. In Poland, all the tests we asked about are available under the compulsory public health insurance and do not require patient co-payment. Most of the tests are dedicated to certain risk groups or patients with specific symptoms. For all of the tests, patients are required to obtain a referral from a GP or a specialist physician. It was found in a nation-wide study on a representative group of adult Poles that in the first half of 2018 alone, as many as 29% of the respondents used publicly funded diagnostic tests (e.g., ultrasound, CT scan, upper endoscopy). What we found worrying in our study group was not only the smaller proportions of testers, but also the fact that big proportions of test users received the tests rather infrequently. For example, in about a half of all the patients who underwent abdominal ultrasound, tests were performed less often than once in 5 years. At this frequency, the chances of diagnosing abdominal aortic aneurysm or kidney cancer at an early stage are slim [44].

The least common test among our participants was dermatoscopy, performed in only 7.38% of the whole group and in 3.1% of farmers, whose sun exposure and skin cancer risk are high [45,46,47]. Unfortunately, the available research suggests that about one-third of farmers may need additional evaluation due to identification of a concerning lesion as a result of screening [47]. Since farmers’ sun protection practices tend to be unsatisfactory [45,47,48], it seems legitimate to implement targeted educational interventions and screening programmes in this risk group [47,48], especially as skin cancer prevention and screening may be uncommon in GP practice [49].

Our results also show that not every woman of appropriate age for cervical and breast cancer screening had ever taken part in it, and many did not participate often enough. For example among the female participants of our study, 75% had ever had a smear test, but only 74.3% of those had been given the test at least once every 3 years. The test could be performed in PHC offices and the acceptance for such practices among Polish patients was found to be high; unfortunately, it hardly ever happens in such settings [50]. Wielkopolskie in 2017–2018 was actually the only province in Poland with an increase in cervical cancer deaths in young women and with a relatively high death rate in the country [3].

The two breast cancer screening tests, namely mammography as a primary method and breast ultrasound as a complementary one, were also underused in our group. Among women over 50 years, 80.9% had mammography at least once but only 68.3% of these had it at least once every 2 years. Among all female participants, 43.2% had a breast ultrasound, with 64.3% of these having it at least once every 2 years. In addition, over one-third of our female respondents (35.2%) never had a breast imaging test, while one in six (18.2%) neither had any imaging tests nor did self-exams. It seems that many of these women are not breast-aware, and consequently may miss a chance to deal with disturbing symptoms early enough. In an attempt to compare our results with other findings from the region, we looked at a study with participants who came from big cities and suburban areas of Wielkopolskie province. As many as 87.2% of its participants had a smear test at least once every 2 years, 66% had breast ultrasound at least once every 2 years, and 87.4% had mammography at least once in a lifetime [51]. According to a larger national study, a smear test was performed at least once in 85% of Polish women, a breast ultrasound in 49%, and mammography in 83% of women aged 50 and above [52]. Our results suggest that women living in rural areas away from big cities take part in cancer screening less often than the general female population.

There is no consensus among various organisations when it comes to clinical recommendations on the need for performing breast self-exams; however, it is recommended that women be educated on the need for looking out for breast changes that may be early warning signs of cancer and should be reported to a physician, especially before a woman reaches the age making her eligible for mammography [53]. In our study group, 63.6% of women said that they performed breast self-exams but most of them did it rarely, while 36.4% never performed them. In contrast, 86.2% of the women from urban and suburban areas in Wielkopolskie province surveyed by Stanisławska et al. reported performing self-exams, although the details of frequency were not provided [51]. Our data are alarming, especially in light of the breast cancer fatality rate in the years 2017–2018—Wielkopolskie province had the highest rate in the country and was one of the two provinces where rural inhabitants had higher rates than city dwellers [3].

These findings suggest profound health inequalities between different parts of Wielkopolskie province, confirming our choice of the target location for the educational workshops on the European Code Against Cancer.

### 4.4. Access to Services

Independently of any socioeconomic factors we analysed, the main reasons for screening non-attendance were a lack of time, lack of need or feeling healthy. The lack of time was also listed in the EU-SILC study as the third most common reason for unmet medical care needs in the EU [54]. This might result from the low priority that respondents give to secondary prevention or from their low health literacy, i.e., their limited ability to obtain, understand, process, and apply health-related information to health decision-making. Health literacy was found to be associated with cancer prevention behaviours [55].

The next most common reason for non-attendance among our respondents was long waiting times to see specialists (17.2%), followed by fear of costs (9%) and GPs’ reluctance to refer patients for screening tests (8.2%). All of these reasons were also found to play a role in non-attendance in a 2017 national survey [52]. Although we did not find a statistically significant association between the fear of costs and age, we did observe a tendency for such fear to be more often reported by older respondents.

In our study, we decided to find out how our respondents dealt with the limited access to care by financing diagnostic tests out of pocket. The tests frequently financed this way were thyroid ultrasound, smear tests, breast ultrasound, dermatoscopy, abdominal ultrasound, and upper endoscopy tests. It should be noted that not only did the respondents perform most of the tests less often than the general population, but they also financed the diagnostics out of pocket more frequently. It seems that even patients who inhabit a socioeconomically deprived region make out-of-pocket payments more often than one might expect [56]. An important message comes from analysing the proportions of patients who paid for particular tests out of pocket. While mammography, FOBT, and chest X-rays rarely became out-of-pocket expenses, almost 60% of thyroid ultrasounds and nearly half of smear tests were financed this way. Our results corroborate earlier findings that a large share—in some regions up to 50%—of all smear tests performed in Poland are financed out of pocket [50,57]. Additionally, in just the first half of 2018, as many as 13% of Polish adults paid for diagnostic tests out of pocket, mostly because of shorter waiting times in the private sector [58]. Although some studies have reported that the share of out-of-pocket payments in Poland fell considerably [13,54], we still found many people paying for private care despite being entitled to free public services, with the main reason being poor access to public care, particularly in the outpatient sector [56]. Similarly, Polish cancer patients complained about difficulties accessing cancer care, especially about long waiting times for diagnostic tests, doctor’s appointments, and treatment. One in three cancer patients reported having paid for some diagnostic examinations [59]. Our findings regarding test use patterns and reported financing methods suggest that patients become well aware of long waiting times at the stage of cancer screening. Another conclusion we might draw is that the district described here and those in similar remote areas are faced with other system-related challenges. These include the scarcity of local PHPs [11,12], as well as a failure of local primary care providers to recruit patients to certain national and regional cancer prevention PHPs (e.g., to a head and neck cancer programme or skin cancer programme) [2,10,12] and to other health prevention programmes such as the pilot programme of coordinated primary care [60]. It seems that the available European structural and investment funds, which usually finance such initiatives, do not always suffice to solve such problems in deprived areas [1]. Additionally, although local authorities can apply for additional funds from the central budget to increase the supply of the least accessible health services, they hardly ever use this opportunity [61]. Only 4 local governments from Wielkopolska region received such support in 2020 [62]. It should be pointed out that the responsibility for health care provision is shared in Poland between the Ministry of Health and local governments, with the latter being responsible for PHC [1].

Our respondents’ health system needs seem to be pointing clearly to the weaknesses of the public system. The needs listed the most often were: better access to physicians, including specialists (65.6%); free screening tests (54.9%); health-related classes or workshops (23.8%); as well as PHPs (22.1%). Indeed, Polish patients were found to be the least satisfied with the access to outpatient physician services among six Central and Eastern European countries that had experienced similar healthcare reforms [63]. Health staff in Poland are scarce; the shortage is one of the most acute in the EU, and Wielkopolska is the region facing the lowest health personnel density [1,22]. These findings do not offer much hope, given the unfavourable epidemiological rates in the region [3]. Low density levels of outpatient doctors can be especially observed in rural areas. While 40% of Polish population live in the country, only 22.4% of public outpatient facilities are located there [23]. Previous research studies have demonstrated that limited access to health services has a negative impact on rural residents’ perceptions of their health [20]. That could partly explain relatively low ratings of our participants’ health status. Moreover, family physicians, who are among the most accessible care providers within the system, have limited possibilities of referring their patients to specialised care owing to insufficient financial resources of the NFZ [1]. This is one of the reasons why stop-gap countermeasures are sometimes implemented, such as the pilot coordinated care programme that enables GPs to provide patients with a wider array of diagnostic tests [6], or limited-scale health interventions that improve access to care in selected rural districts [64].

### 4.5. Healthcare Staff and a Key Role of GPs as Health Promoters

We believe that it is a crucial role of healthcare staff to inform patients that thanks to PHPs, disease prevention delivered to specific risk groups does not entail long waiting times and out-of-pocket spending. It is also health professionals’ responsibility to provide clear information on the conditions under which particular screening tests are available within the public health system so as to eliminate the cost barrier for patients. Admittedly, the reluctance of our respondents GPs’ to refer patients for diagnostic tests was not the most important barrier to screening participation; however, other studies have shown that family physicians’ counselling to encourage participation in secondary prevention may become a decisive incentive for high-risk patients [65] and seems to be among their most important responsibilities towards local populations [5,40,66]. This is why we checked how many of our respondents who participated in cancer screening had ever been suggested by their GPs to undergo particular tests. The biggest proportion was found among the patients who had received a colonoscopy (60%). The importance of GPs in convincing patients to undertake this test was also found in another study [65]. This is important, finding especially in Wielkopolskie province, where the risk of developing cancer (colorectal cancer in particular) is the highest in Poland [3]. Unfortunately, we also found that female patients were seldom recommended to take smear tests (28.8%) or various forms of breast exams (around 40%). Similar results were reported in two other studies [42,67]. It appears that healthcare professionals do not sufficiently engage in preventive counselling [68,69,70], and that their cancer prevention cancelling needs improvement [42,71]. Unsurprisingly, 54.9% of our respondents listed the need for promoting free screening tests among their most important unmet healthcare needs.

One earlier study suggested that population-based programmes should involve all healthcare providers that may come into contact with a target population, referring in particular to primary care physicians [40]. As GPs are usually the doctors that patients reach first and have long-term relationships with, they may play a fundamental role in promoting screening tests [40,72,73,74]. Primary care facilities may also effectively mitigate health inequalities [70]. Such facilities are visited by as many as three-quarters of Poles in just six months [58], so their potential influence is enormous. It was demonstrated that better knowledge of the methods of early cancer detection is associated with wider use of cancer screening [75]. Research studies have also shown that a more active approach of PHC results in increased cancer screening participation [72,74]. Although the role of primary care practitioners in cancer screening varies depending on the country, type of cancer, selected strategy, and local health policy [76], it seems that Polish GPs should become more involved in patient recruitment. What might motivate them to do so is the prospective system of financial incentives provided to those GPs who are successful at increasing screening uptake [70,76,77]. Another solution could be to launch a system reminding doctors to refer patients for screening tests [71]. In addition, the importance of secondary prevention should be repeatedly emphasised during university and continuing education of health personnel [70,78]. We agree with European primary care practitioners that the struggle against cancer could become more effective through the simultaneous use of a number of strategies, by educating patients, strengthening the education of health staff, enhancing collaboration within the health system, improving access to health services and diagnostic tests in PHC, promoting the use of IT solutions, and better financing [66]. In our opinion, health inequalities cannot be eliminated through declarations alone. Countermeasures must be implemented first in neglected regions such as the one we are describing here. National health reports, novel public health tools, and field studies such as ours can show the areas with the most pressing needs.

### 4.6. Study Strengths and Limitations

Our study has a few limitations. Firstly, our respondents were the participants of open educational workshops who signed up themselves. Although the workshops had been widely advertised and organised in such a way as to create equal chances for everyone to take part, the recruitment process and the resulting sample cannot be deemed to be representative of the population. The strength of our project was the fact that we did not just explore the determinants of health inequalities in our study group, but we also tried to reduce them—we not only talked, but also acted. Despite our limited resources and tight budget, we tried to do our best under the circumstances. As our next step, we are now showing the areas for future research and recommendations for more effective health interventions and policies aiming to ameliorate health inequalities.

Secondly, our results on participation in screening tests and family history of cancer are based on self-reported data. We obtained patients’ opinions, but we neither asked physicians nor checked patients’ medical records, so the results may be subject to recall bias. Additionally, female participants were overrepresented in our group, which may have resulted in bigger test participation rates than those we would observe in a more gender-balanced study group.

Thirdly, we did not collect extensive data on social determinants of health in our study group. Nevertheless, we tried to describe some of those determinants using the available research in order to present the wider context.

## 5. Conclusions

This study is a voice in the debate on health inequalities and on the necessity to intensify health promotion in rural, remote, and deprived populations. Our findings suggest that despite the best intentions of policymakers, there is still a high risk of unmet health needs in neglected areas such as the one described here. Although many health determinants—for instance people’s education or economic status—cannot be changed easily, some factors—for example access to health care and health literacy—could be improved.

Our study provides information that could help health professionals working in occupational healthcare and in rural clinics to take better care of disadvantaged rural populations. This is why we provided our findings to both the local PHC facilities and to the authorities of the commune where the CBPR took place. Our conclusions may be relevant not only to the region described here, but also to other regions with similar conditions. In our opinion, the following steps could be taken to redress health inequalities in deprived rural areas:

- The central or provincial governments could support the local authorities in developing local health policies through both financing and training;
- PHC personnel could use their high status in rural populations and their position of health system gatekeepers responsibly, remembering that it largely depends on them if and how their patients access and use health services;
- University staff could translate theoretical knowledge into practice by becoming more involved in field work and engage local communities in the area of health promotion in CPBR projects;
- The findings from field studies regarding health needs and inequalities should be more intensely publicised and discussed among the key players of the health system, who should be held responsible for sharing experiences and helping to develop, finance, and implement good practices.

If we want to materially mitigate health inequalities, we need to translate the long-proposed public health strategies into specific viable interventions.

## Figures and Tables

**Table 1:** Sociodemographic status of the study group (n = 122).

Variable		n	%	Education (%)					Economic Status (%)				Occupation (%)					
				Primary	Lower Secondary	Vocational	Secondary	Higher	Bad	Average	Good	Very Good	Farmers	Other Manual Workers	Office Workers or Other Specialists	Health Professionals	Teachers/Educators	Unemployed
**Total**		122	100	17.2	1.6	26.2	35.2	19.7	4.1	32.8	58.2	4.9	53.3	16.4	10.7	6.5	5.7	7.4
**Gender**	F	88	72.1	14.8	2.3	21.6	42	19.3	2.3	33	60.2	4.5	51.1	11.3	13.6	8	8	8
	M	34	27.9	23.5	0	38.2	17.6	20.6	8.8	32.3	52.9	5.9	58.8	29.4	2.9	2.9	0	5.9
**Age**	<25	9	7.3	0	22.2	22.2	44.5	11.1	0	33.3	55.6	11.1	0	33.3	11.1	0	0	55.6
	25–44	39	32	5.1	0	18	28.2	48.7	7.7	12.8	74.4	5.1	43.6	25.7	5.1	5.1	15.4	5.1
	45–64	50	41	22	0	26	44	8	2	38	54	6	66	8	10	10	2	4
	65 or older	24	19.7	33.3	0	41.7	25	0	4.2	54.2	41.7	0	62.5	12.5	20.8	4.2	0	0
**Professional status**	employed	67	54.9	10.5	0	25.4	31.3	32.8	3	23.9	68.6	4.5	49.3	23.9	8.9	8.9	8.9	0
	retired	46	37.7	28.3	0	28.3	39.1	4.3	4.3	43.5	50	2.2	69.6	8.7	15.2	4.3	2.2	0
	unemployed	4	3.3	25	0	50	25	0	25	25	25	25	0	0	0	0	0	100
	students	5	4.1	0	40	0	60	0	0	60	20	20	0	0	0	0	0	100

Note: F—female; M—male.

**Table 2:** BMI and self-perceived health (n = 122).

Variable		n	BMI		Self-Perceived Health (%)					
			% of Normal Results	*p*	Very Bad	Bad	Fair	Good	Very Good	*p*
**Total**		122	37.7		0.8	4.1	49.2	36.9	9	
**Age**	<25	9	88.9	<0.0001 ^a^	0	0	11.1	33.3	55.6	<0.0001 ^d^ r = −0.55
	25–44	39	56.4		0	5.1	23.1	59	12.8	
	45–64	50	20		2	2	58	36	2	
	65 or older	24	25		0	8.3	87.5	4.2	0	
**Gender**	F	88	38.6	0.7327 ^b^	0	4.5	50	36.4	9.1	0.8459 ^a^
	M	34	35.3		2.9	2.9	47.1	38.2	8.8	
**Education**	primary	21	28.6	0.1404 ^a^	4.8	4.8	76.2	14.3	0	0.0034 ^d^ r = 0.26
	lower secondary	2	100		0	0	0	0	100	
	vocational	32	28.1		0	3.1	56.3	31.3	9.4	
	secondary	43	37.2		0	4.7	44.2	44.2	7	
	higher	24	54.2		0	4.2	29.2	54.2	12.5	
**Professional status**	employed	67	46.3	0.0002 ^c^	0	1.5	35.8	52.2	10.4	<0.0001 ^e^
	retired	46	19.6		2.2	6.5	76.1	15.2	0	
	unemployed	4	25		0	25	25	25	25	
	students	5	100		0	0	0	40	60	
**Occupation**	farmers	65	32.3	0.1753 ^c^	1.5	1.5	61.5	29.2	6.2	0.0605 ^e^
	other manual workers	20	45		0	0	35	55	10	
	office workers or other specialists	13	38.5		0	15.4	38.5	46.2	0	
	health professionals	8	12.5		0	12.5	37.5	50	0	
	teachers/educators	7	57.1		0	0	57.1	28.6	14.3	
	unemployed	9	66.7		0	11.1	11.1	33.3	44.4	
**Economic status**	bad	5	40	0.6431 ^a^	0	0	100	0	0	0.0002 ^d^ r = 0.33
	average	40	32.5		2.5	5	62.5	27.5	2.5	
	good	71	40.8		0	4.2	39.4	46.5	9.9	
	very good	6	33.3		0	0	33.3	16.7	50	
**Attitude to screening**	does not attend	60	50	0.0013 ^b^	1.7	3.3	35	45	15	0.0055 ^a^
	attends	53	20.8		0	5.7	64.2	26.4	3.8	
**Family history of cancer**	no	45	45.2	0.2991 ^b^	2.4	0	47.6	40.5	9.5	0.5996 ^a^
	yes	66	35.3		0	5.9	50	35.3	8.8	

Note: n—group size; M—mean; ^a^ Chi-squared test for trends; ^b^ Pearson’s chi-squared test; ^c^ Fisher’s exact test; ^d^ Spearman’s rank correlation coefficient; ^e^ Kruskal–Wallis ANOVA.

**Table 3:** The reported cancer screening attendance (n = 122) and the main reasons for non-attendance (n = 60).

Variable		n	Reported Cancer Screening Attendance (%)			Main Reasons for Non-Attendance among the Non-Attending Participants (%)							
			No	Yes	*p*	Lack of Time, Lack of Need, Feeling Healthy	*p*	GPs’ Reluctance to Refer Patients for Screening Tests	*p*	Long Waiting Times to See Specialists	*p*	Fear of Costs	*p*
**Total**		122	49.2	43.4		32.8		8.2		17.2		9	
**Age**	<25	9	88.9	0	<0.0001 ^a^	55.6	0.3101 ^a^	11.1	0.279 ^a^	11.1	0.2391 ^a^	0	0.0901 ^a^
	25–44	39	71.8	20.5		64.5		22.6		32.3		16.1	
	45–64	50	38	54		54.2		8.7		34.8		17.4	
	65 or older	24	20.8	75		33.3		0		40		40	
**Gender**	F	88	42	52.3	0.0025 ^b^	59.5	0.622 ^b^	14.6	1 ^c^	34.1	0.4728 ^b^	19.5	0.5059 ^c^
	M	34	67.6	20.6		53.6		14.8		25.9		11.1	
**Education**	primary	21	42.9	52.4	0.6507 ^a^	55.6	0.4864 ^a^	0	0.0222 ^a^	22.2	0.2796 ^a^	22.2	0.9627 ^a^
	lower secondary	2	100	0		0		0		0		0	
	vocational	32	53.1	37.5		57.1		10		25		15	
	secondary	43	41.9	48.8		59.1		13.6		40.9		13.6	
	higher	24	58.3	37.5		62.5		33.3		33.3		20	
**Professional status**	employed	67	55.2	34.3	0.0005 ^c^	66.7	0.8872 ^b^	18.2	0.395 ^c^	31.8	0.0655 ^b^	18.2	0.2549 ^c^
	retired	46	32.6	65.2		31.3		13.3		40		20	
	unemployed	4	100	0		75		0		0		0	
	students	5	80	0		40		0		20		0	
**Occupation**	farmers	65	43.1	50.8	<0.0001 ^c^	51.5	0.8872 ^c^	21.9	0.395 ^c^	46.9	0.0655 ^c^	15.6	0.2549 ^c^
	other manual workers	20	75	15		64.7		11.8		11.8		17.6	
	office workers or other specialists	13	46.2	46.2		57.1		0		33.3		16.7	
	health professionals	8	0	100		0		0		0		0	
	teachers/educators	7	42.9	42.9		75		25		25		50	
	unemployed	9	88.9	0		55.6		0		11.1		0	
**Economic status**	bad	5	100	0	0.2026 ^a^	60	0.4367 ^a^	0	0.1023 ^a^	0	0.6425 ^a^	20	0.946 ^a^
	average	40	45	37.5		64		4.2		37.5		16.7	
	good	71	46.5	50.7		52.8		25.7		31.4		14.3	
	very good	6	66.7	33.3		50		0		25		25	
**Family history of cancer**	no	45	59.5	38.1	0.0585 ^b^	42.3	0.1958 ^c^	11.5	0.7153 ^c^	23.1	0.389 ^c^	15.4	0.6906 ^c^
	yes	66	38.2	52.9		60.6		16.1		35.5		9.7	

Note: n—group size; ^a^ Chi-squared test for trend; ^b^ Pearson’s chi-squared test; ^c^ Fisher’s exact test.

**Table 4:** The respondents’ participation in selected diagnostic tests ^1^.

Diagnostic Test	Participants Who Ever Had the Test (%)	Participants Who Had the Test and Whose GPs Suggested It (%)	Test Financing (%)		Frequency of Testing (%)							Reason for Testing (%)			
			Public	Out-of-Pocket Only	1 × Year	1 × 2 Years	1 × 3 Years	1 × 4 Years	1 × 5 Years	Less Often	1 × A Lifetime	Check-Up/Prevention	Confirming a Diagnosis	Ailment/Pain	Other
**Chest X-ray**	65.6	57.5	92.5	7.5	5.1	11.4	11.4	6.3	6.3	32.9	25.3	37.0	29.6	21	7.4
**Abdominal ultrasound**	54.1	65.2	69.7	30.3	7.6	16.7	7.6	4.6	12.1	18.2	31.8	30.4	29	39.1	1.5
**Thyroid ultrasound**	25.4	61.3	41.9	58.1	22.6	12.9	6.5	3.2	0	12.9	38.7	45.2	32.3	16.1	3.2
**Upper endoscopy**	25.4	58.1	71	29	0	3.2	9.7	6.5	9.7	22.6	48.4	22.6	22.6	51.6	3.2
**Colonoscopy**	20.5	60	84	16	4	0	0	0	32	16	48	48	12	36	4
**FOBT**	11.5	42.9	92.9	7.1	0	0	7.1	0	21.4	14.3	50	71.4	7.1	14.3	7.1
**Tumour markers**	7.4	44.4	66.7	33.3	22.2	11.1	11.1	0	0	22.2	22.2	44.4	44.4	11.1	0
**Dermatoscopy**	7.4	66.7	66.7	33.3	11.1	0	11.1	0	0	33.3	44.4	60	30	10	0
PSA *	32.4	72.7	81.8	18.2	9.1	0	18.2	0	27.3	9.1	36.4	54.6	36.4	9.1	0
Smear test **	75	28.8	51.5	48.5	15.2	39.4	19.7	6.1	1.5	12.1	4.6	82.6	4.4	10.1	0
Mammography **	80.9	41.5	98	2	2.4	65.9	14.6	2.4	4.9	4.9	4.9	95.1	2.4	2.4	0
Breast ultrasound **	43.2	42.1	63.2	36.8	2.6	23.7	15.8	2.6	10.5	21.1	21.1	79.5	10.3	15.4	0

Note: * in men, ** in women; mammography in women aged 50 or older; ^1^ in the rare cases where the respondents were unable to recall the frequency or reason, the results do not add up to 100%; FOBT—fecal occult blood test; PSA—prostate-specific antigen level test.

## Data Availability

The anonymised data presented in this study are available on request from the corresponding author.

## Abbreviations

BMI	body mass index
CanCon	Joint Action on Cancer Control
CBPR	community-based participatory research
GP	general practitioner
iPAAC	Innovative Partnership for Action Against Cancer
NFZ	National Health Fund (Polish: Narodowy Fundusz Zdrowia)
PHC	primary healthcare
PHP	public health programme
WHO	World Health Organization

## References

[1] OECD/European Observatory on Health Systems and Policies (2019). Poland: Country Health Profile 2019.

[2] Wojtyniak B., Goryński P. (2018). Sytuacja Zdrowotna Ludności Polski i jej Uwarunkowania [Health Status of Polish Population and Its Determinants].

[3] Wojtyniak B., Goryński P. (2020). Health Status of Polish Population and Its Determinants.

[4] Schüz J., Espina C., Villain P., Herrero R., Leon M.E., Minozzi S., Romieu I., Segnan N., Wardle J., Wiseman M. (2015). European code against cancer 4th edition: 12 ways to reduce your cancer risk. Cancer Epidemiol..

[5] (2017). Ustawa z dnia 27 października 2017 r. o podstawowej opiece zdrowotnej Dz. U. z 2017 r. poz. 2217 [Act of 27 October 2017 on Primary Health Care]. J. Laws.

[6] Karasiewicz M., Chawłowska E.M., Lipiak A., Staszewski R. (2020). A Polish pilot programme of coordinated care: A herald of change or a missed opportunity? A critical debate. Front. Public Health.

[7] (2016). Rozporządzenie Rady Ministrów z dnia 4 sierpnia 2016 r. w sprawie Narodowego Programu Zdrowia na lata 2016–2020 Dz.U. 2016 poz. 1492 [Regulation on the National Health Programme for the Years 2016–2020 Issued by the Council of Ministers on 4 August 2016]. J. Laws.

[8] (2021). Rozporządzenie Rady Ministrów z dnia 30 marca 2021 r. w sprawie Narodowego Programu Zdrowia na lata 2021–2025 Dz. U. 2021 poz. 642 [Regulation on the National Health Programme for the Years 2021–2025 Issued by the Council of Ministers on 30 March 2021]. J. Laws.

[9] (2019). Ustawa z dnia 26 kwietnia 2019 r. o Narodowej Strategii Onkologicznej Dz. U. 2019 poz. 969 [Act of 26 April 2019 on the National Cancer Control Strategy]. J. Laws.

[10] Ministerstwo Zdrowia/Narodowy Fundusz Zdrowia (Ministry of Health/National Health Fund) Programy Profilaktyczne [Public Health Programmes]. https://www.nfz.gov.pl/dla-pacjenta/programy-profilaktyczne/.

[11] Agencja Oceny Technologii Medycznych i Taryfikacji (The Agency for Health Technology Assessment and Tariff System) Biuletyn Informacji Publicznej Agencji Oceny Technologii Medycznych i Taryfikacji [The Newsletter of the Agency for Health Technology Assessment and Tariff System]. http://bipold.aotm.gov.pl/index.php/repozytorium-ppz-2/opinie-o-projektach-programow-zdrowotnych-realizowanych-przez-jst.

[12] Ministerstwo Zdrowia (Ministry of Health) Baza Analiz Systemowych i WdrożEniowych. [The Database of System and Implementation Analyses]. https://basiw.mz.gov.pl/index.html#/visualization?id=3408.

[13] Tambor M., Klich J., Domagała A. (2021). Financing healthcare in central and eastern european countries: How far are we from universal health coverage?. Int. J. Environ. Res. Public Health.

[14] Eikemo T.A., Huisman M., Bambra C., Kunst A.E. (2008). Health inequalities according to educational level in different welfare regimes: A comparison of 23 European countries. Sociol. Health Illn..

[15] Hoffmann R., Eikemo T.A., Kulhánová I., Dahl E., Deboosere P., Dzúrová D., van Oyen H., Rychtaríková J., Strand B.H., Mackenbach J.P. (2013). The potential impact of a social redistribution of specific risk factors on socioeconomic inequalities in mortality: Illustration of a method based on population attributable fractions. J. Epidemiol. Community Health.

[16] Eikemo T.A., Bambra C., Huijts T., Fitzgerald R. (2017). The First Pan-European Sociological Health Inequalities Survey of the General Population: The European Social Survey Rotating Module on the Social Determinants of Health. Eur. Sociol. Rev..

[17] Van de Velde S., Bracke P., Levecque K. (2010). Gender differences in depression in 23 European countries. Cross-national variation in the gender gap in depression. Soc. Sci. Med..

[18] Binkowska-Bury M., Iwanowicz-Palus G., Kruk W., Perenc L., Mazur A., Filip R., Januszewicz P. (2016). Pro-health behaviours—A sense of coherence as the key to a healthy lifestyle in rural areas?. Ann. Agric. Environ. Med..

[19] Pinidiyapathirage J., O’Shannessy M., Harte J., Brumby S., Kitchener S. (2018). Chronic disease and health risk behaviors among rural agricultural workforce in Queensland. J. Agromed..

[20] Laskowska I. (2015). Availability of health services vs. Health condition of residents of rural areas in Poland—Analysis performed on the basis of EHIS 2009. Ann. Agric. Environ. Med..

[21] Pantyley V. (2017). Health inequalities among rural and urban population of eastern Poland in the context of sustainable development. Ann. Agric. Environ. Med..

[22] Winkelmann J., Muench U., Maier C.B. (2020). Time trends in the regional distribution of physicians, nurses and midwives in Europe. BMC Health Serv. Res..

[23] Bendowska E., Borawska M., Jankowski Ł., Kalski J., Lech I., Medolińska K., Pytalska A., Stawikowska M., Wielechowska K., Zych M. (2020). Obszary wiejskie w Polsce w 2018 r. [Rural Areas in Poland in 2018].

[24] Zahnd W.E., Askelson N., Vanderpool R.C., Stradtman L., Edward J., Farris P.E., Petermann V., Eberth J.M. (2019). Challenges of using nationally representative, population-based surveys to assess rural cancer disparities. Prev. Med..

[25] Nowicki G.J., Ślusarska B., Piasecka H., Bartoszek A., Kocka K., Deluga A. (2018). The status of cardiovascular health in rural and urban areas of Janów Lubelski district in eastern Poland: A population-based study. Int. J. Environ. Res. Public Health.

[26] Szeszenia-Dąbrowska N., Świątkowska B., Wilczyńska U. (2016). Occupational diseases among farmers in Poland. Med. Pr..

[27] Smetkowski M., Gorzelak G., Płoszaj A., Rok J. (2015). Powiaty Zagrożone Deprywacją: Stan, Trendy i Prognoza [Poviats Threatened by Deprivation: State, Trends and Prospects].

[28] Narodowy Fundusz Zdrowia (National Health Programme) Dane o Realizacji Programów [The Attendance Rates of Public Health Programmes]. https://www.nfz.gov.pl/dla-pacjenta/programy-profilaktyczne/dane-o-realizacji-programow/.

[29] Kałużny D., Paczesna A., Jarońska D. (2017). Diagnoza Zjawisk Kryzysowych dla Miasta i Gminy Dąbie [The Diagnosis of Critical Phenomena Performed for Dąbie Town and Commune].

[30] Urząd Marszałkowski Województwa Wielkopolskiego [The Marshal Office of the Wielkopolska Region] (2015). Wielkopolski Regionalny Program Operacyjny na Lata 2014–2020. Szczegołówy Opis Osi Priorytetowych Programu Operacyjnego (Uszczegółowienie WRPO 2014+). Wersja 1.8. [Regional Operational Programme for the Wielkopolska Region for the Years 2014–2020. A Detailed Description of Priority Axes of the Operational Programme (ROPWR 2014+ Specification). Version 1.8.].

[31] Polska w Liczbach: Gmina Dąbie (Wielkopolskie) w Liczbach [Poland in Numbers: Dąbie Commune, Wielkopolskie, in Numbers]. https://www.polskawliczbach.pl/gmina_Dabie_wielkopolskie.

[32] Urząd Statystyczny w Poznaniu (Statistical Office in Poznań) Statystyczne Vademecum Samorządowca [A Statistical Handbook for Local Government Officials]. https://poznan.stat.gov.pl/statystyczne-vademecum-samorzadowca/.

[33] World Health Organization Commission on Social Determinants of Health. https://www.who.int/teams/social-determinants-of-health/equity-and-health/commission-on-social-determinants-of-health.

[34] Innovative Partnership for Action against Cancer iPAAC Contest of Best Practices Tackling Social Inequalities in Cancer Prevention. https://www.ipaac.eu/en/contest-best-practices/.

[35] Peiró Pérez R., Barceló A.M., De Lorenzo F., Spadea T., Missinne S., Florindi F., Zengarini N., Apostolidis K., Coleman M.P., Allemani C. (2017). Policy Paper on Tackling Social Inequalities in Cancer Prevention and Control for the European Population. https://cancercontrol.eu/archived/uploads/PolicyPapers27032017/Policy_Paper_4_Tackling.pdf.

[36] Kasa Rolniczego Ubezpieczenia Społecznego (Agricultural Social Insurance Fund) Wstępny Bilans Unikatowych Programów Profilaktyki Zdrowia z Udziałem KRUS [Preliminary Findings from Unique Public Health Programmes Cofinanced by ASIF]. https://www.krus.gov.pl/aktualnosci/dokument/artykul/wstepny-bilans-unikatowych-programow-profilaktyki-zdrowia-z-udzialem-krus/.

[37] Eurostat Self-Perceived Health by Sex, Age and Income Quintile (HLTH_SILC_10). https://ec.europa.eu/eurostat/databrowser/view/hlth_silc_10/default/table?lang=en.

[38] Główny Urząd Statystyczny (Central Statistical Office) Stan Zdrowia Ludności Polski w 2014 r. [Health Status of Population in Poland in 2014.]. https://stat.gov.pl/obszary-tematyczne/zdrowie/zdrowie/stan-zdrowia-ludnosci-polski-w-2014-r-,6,6.html.

[39] Bixby H., Bentham J., Zhou B., Di Cesare M., Paciorek C.J., Bennett J.E., Taddei C., Stevens G.A., Rodriguez-Martinez A., Carrillo-Larco R.M. (2019). Rising rural body-mass index is the main driver of the global obesity epidemic in adults. Nature.

[40] Gianino M.M., Lenzi J., Bonaudo M., Fantini M.P., Siliquini R., Ricciardi W., Damiani G. (2018). Organized screening programmes for breast and cervical cancer in 17 EU countries: Trajectories of attendance rates. BMC Public Health.

[41] Hocaoglu M., Adeviye Ersahin A., Akdeniz E. (2017). Evaluation on the practice and behaviour of women applied for gynecology outpatient clinics about screening methods for early diagnosis of breast cancer. Eur. J. Breast Health.

[42] Dyzmann-Sroka A., Bagniewska K., Chyła K., Dec K., Dominiak J., Dubnowska M., Fabian D., Fajge A., Fedon K., Foryszewska P. (2012). Dlaczego Wielkopolanki nie robią badań mammograficznych?—Raport [Women in the Wielkopolska Region, Why Don’t They Take Mammography?]. Zesz. Nauk. WCO Lett. Oncol. Sci..

[43] Shah M., Zhu K., Palmer R.C., Jatoi I., Shriver C., Wu H. (2007). Breast, colorectal, and skin cancer screening practices and family history of cancer in U.S. women. J. Women’s Health.

[44] Rossi S.H., Klatte T., Usher-Smith J., Stewart G.D. (2018). Epidemiology and screening for renal cancer. World J. Urol..

[45] Moeini B., Ezati E., Barati M., Rezapur-Shahkolai F., Mohammad Gholi Mezerji N., Afshari M. (2019). Skin cancer preventive behaviors in Iranian farmers: Applying protection motivation theory. Work. Health Saf..

[46] Kachuri L., Harris M.A., MacLeod J.S., Tjepkema M., Peters P.A., Demers P.A. (2017). Cancer risks in a population-based study of 70,570 agricultural workers: Results from the Canadian census health and Environment cohort (CanCHEC). BMC Cancer.

[47] Carley A., Stratman E. (2015). Skin cancer beliefs, knowledge, and prevention practices: A comparison of farmers and nonfarmers in a Midwestern population. J. Agromed..

[48] Kearney G.D., Xu X., Balanay J.A.G., Becker A.J. (2014). Sun safety among farmers and farmworkers: A review. J. Agromed..

[49] Rat C., Houd S., Gaultier A., Grimault C., Quereux G., Mercier A., Letrilliart L., Dreno B., Nguyen J.M. (2017). General practitioner management related to skin cancer prevention and screening during standard medical encounters: A French cross-sectional study based on the International Classification of Primary Care. BMJ Open.

[50] Nessler K., Chan S.K.F., Ball F., Storman M., Chwalek M., Krztoń-Królewiecka A., Kryj-Radziszewska E., Windak A. (2019). Impact of family physicians on cervical cancer screening: Cross-sectional questionnaire-based survey in a region of southern Poland. BMJ Open.

[51] Stanisławska J., Janikowska K., Stachowska M., Talarska D., Drozd-Gajdus E., Szewczyczak M. (2016). Ocena wiedzy kobiet w zakresie profilaktyki raka piersi i raka szyjki macicy [Assessment of women’s knowledge on prevention of breast cancer and cervical cancer]. Probl. Hig. Epidemiol..

[52] Kantar Millward Brown, Ministerstwo Zdrowia (2017). Badanie Postaw Wobec Zachowań Zdrowotnych w Zakresie Profilaktyki Nowotworowej Wśród Mieszkańców Polski ze Szczególnym Uwzględnieniem Postaw Polek Wobec Raka Szyjki Macicy i Raka Piersi [A Survey of Attitudes to Cancer Prevention Behaviours among Poles, with Particular Emphasis on Attitudes of Polish Women to Cervical Cancer and Breast Cancer].

[53] National Health Service How Should I Check My Breasts?—NHS. https://www.nhs.uk/common-health-questions/womens-health/how-should-i-check-my-breasts/.

[54] Chaupain-Guillot S., Guillot O. (2015). Health system characteristics and unmet care needs in Europe: An analysis based on EU-SILC data. Eur. J. Health Econ..

[55] Fleary S.A., Paasche-Orlow M.K., Joseph P., Freund K.M. (2019). The relationship between health literacy, cancer prevention beliefs, and cancer prevention behaviors. J. Cancer Educ..

[56] Tambor M., Pavlova M., Rechel B., Golinowska S., Sowada C., Groot W. (2014). The inability to pay for health services in Central and Eastern Europe: Evidence from six countries. Eur. J. Public Health.

[57] Nowakowski A., Cybulski M., Śliwczyński A., Chil A., Teter Z., Seroczyński P., Arbyn M., Anttila A. (2015). The implementation of an organised cervical screening programme in Poland: An analysis of the adherence to European guidelines. BMC Cancer.

[58] Centrum Badania Opinii Społecznej (Public Opinion Research Center) (2018). Korzystanie ze Świadczeń i Ubezpieczeń Zdrowotnych [Use of Healthcare Services and Insurance].

[59] Osowiecka K., Sroda R., Saied A., Szwiec M., Mangold S., Osuch D., Nawrocki S., Rucinska M. (2020). Patients’ non-medical and organizational needs during cancer diagnosis and treatment. Int. J. Environ. Res. Public Health.

[60] Akademia NFZ/Ministerstwo Zdrowia (NFZ Academy/Ministry of Health) Realizatorzy POZ PLUS [The Providers Implementing POZ PLUS]. https://akademia.nfz.gov.pl/poz-plus-2/realizatorzy-poz-plus/.

[61] (2004). Ustawa z dnia 27 sierpnia 2004 r. o świadczeniach opieki zdrowotnej finansowanych ze środków publicznych Dz. U. 2004 Nr 210 poz. 2135 [Act of 27 August 2004 on Health Care Services Financed from Public Sources]. J. Laws.

[62] Narodowy Fundusz Zdrowia (National Health Fund) Dofinansowanie Programów Polityki Zdrowotnej dla Jednostek Samorządu Terytorialnego w 2020 Roku [Local Public Health Programmes Which Received Support from Central Funds in 2020]. https://www.nfz.gov.pl/biuletyn-informacji-publicznej-wielkopolskiego-ow-nfz/ofinansowanieprogramwpolitykizdrowotnejdlajednosteksamorzduterytorialnegow2020roku/lista-zaakceptowanych-wnioskow,2.html.

[63] Stepurko T., Pavlova M., Groot W. (2016). Overall satisfaction of health care users with the quality of and access to health care services: A cross-sectional study in six Central and Eastern European countries. BMC Health Serv. Res..

[64] Prawo.pl KRUS Realizuje Trzy Programy Profilaktyki Zdrowotnej [ASIF Runs Three Public Health Programmes]. https://www.prawo.pl/zdrowie/krus-realizuje-trzy-programy-profilaktyki-zdrowotnej,245899.html.

[65] Guzek M., Szafraniec-Buryło S., Wyrębiak A., Kowalczyk D., Bukato G., Prusaczyk A., Żuk P., Czech M., Kurpas D. (2019). The role of primary care physicians and nurses in convincing patients to participate in a colorectal cancer screening program in a Polish coordinated care organization: A questionnaire-based study. Med. Sci. Pulse.

[66] Harris M., Thulesius H., Neves A.L., Harker S., Koskela T., Petek D., Hoffman R., Brekke M., Buczkowski K., Buono N. (2019). How European primary care practitioners think the timeliness of cancer diagnosis can be improved: A thematic analysis. BMJ Open.

[67] Poniedziałek B., Rebelka M., Makowska K., Piotrowska J., Karczewski J., Rzymski P. (2014). Do we need to improve breast cancer education? Attitude towards breast self-examination and screening programmes among Polish women. J. Med. Sci..

[68] Znyk M., Polańska K., Wojtysiak P., Szulc M., Bąk-Romaniszyn L., Makowiec-Dąbrowska T., Zajdel-Całkowska J., Kaleta D. (2019). Predictors of counselling related to a healthy lifestyle carried out by a general practitioner. Int. J. Environ. Res. Public Health.

[69] McCall-Hosenfeld J.S., Weisman C.S. (2011). Receipt of preventive counseling among reproductive-aged women in rural and urban communities. Rural Remote Health.

[70] OECD (2020). Realising the Potential of Primary Health Care.

[71] Siembida E.J., Radhakrishnan A., Nowak S.A., Parker A.M., Pollack C.E. (2017). Linking Reminders and physician breast cancer screening recommendations: Results from a national survey. JCO Clin. Cancer Inform..

[72] Emery J.D., Shaw K., Williams B., Mazza D., Fallon-Ferguson J., Varlow M., Trevena L.J. (2014). The role of primary care in early detection and follow-up of cancer. Nat. Rev. Clin. Oncol..

[73] Funston G., O’Flynn H., Ryan N.A.J., Hamilton W., Crosbie E.J. (2018). Recognizing gynecological cancer in primary care: Risk factors, red flags, and referrals. Adv. Ther..

[74] Rubin G., Berendsen A., Crawford S.M., Dommett R., Earle C., Emery J., Fahey T., Grassi L., Grunfeld E., Gupta S. (2015). The expanding role of primary care in cancer control. Lancet Oncol..

[75] Bird Y., Banegas M.P., Moraros J., King S., Prapasiri S., Thompson B. (2011). The impact of family history of breast cancer on knowledge, attitudes, and early detection practices of Mexican women along the Mexico-US border. J. Immigr. Minor. Health.

[76] Mauro M., Rotundo G., Giancotti M. (2019). Effect of financial incentives on breast, cervical and colorectal cancer screening delivery rates: Results from a systematic literature review. Health Policy.

[77] Pochrzęst-Motyczyńska A. Premie finansowe dla lekarzy poz i szpitali—Wywiad z Adamem Niedzielskim, p.o. Prezesa NFZ [Financial Incentives for PHC Doctors and Hospitals: An Interview with Adam Niedzielski]. https://www.prawo.pl/zdrowie/premie-finansowe-dla-lekarzy-poz-i-szpitali-wywiad-z-adamem,485048.html.

[78] Selby K., Bartlett-Esquilant G., Cornuz J. (2018). Personalized cancer screening: Helping primary care rise to the challenge. Public Health Rev..

